# New Substituted Isocoumarins and Dihydroisocoumarins and their Cytotoxic Activities

**DOI:** 10.3797/scipharm.1011-10

**Published:** 2010-12-18

**Authors:** Veronika Hampl, Isolde Wetzel, Franz Bracher, Jürgen Krauss

**Affiliations:** Department of Pharmacy – Center for Drug Research, Ludwig-Maximilians University, Butenandtstr. 5–13, D-81377 Munich, Germany

**Keywords:** Isocoumarins, Dihydroisocoumarins, Sonogashira reaction, Cyclization, Cytotoxicity

## Abstract

New isocoumarins were prepared in an efficient way from 2-iodobenzoic acid derivatives and hept-1-yne in a Sonogashira reaction, followed by spontaneous cyclization. Catalytic hydrogenation gave the corresponding dihydroisocoumarins. A 4-chloroisocoumarin was prepared on an alternative pathway. Some of the new compounds showed moderate cytotoxic activities against a human leukemia cell line (HL 60).

## Introduction

**Fig. 1.**
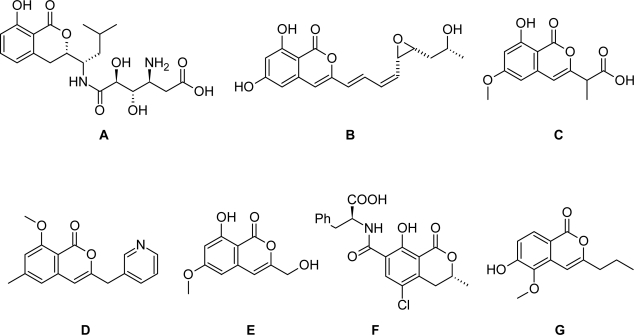
Structures of AI-77-B (**A**), paraphaeosphaerin A (**B**), NM-3 (**C**), 185322 (**D**), cytogenin (**E**), ochratoxin A (**F**), and scoparine A (**G**).

Isocoumarins are a class of natural lactones with high structural diversity exhibiting a broad spectrum of biological activities [1]. Considerable work has been published over decades about their chemistry [2] and biology [3], and a number of natural and synthetic isocoumarins have been shown to exhibit significant cytotoxic and antitumor activities. Among these are numerous isocoumarins bearing various types of substituents (alkyl, alkenyl or aryl groups) at 3-position, as well as the dihydroisocoumarin AI-77-B (**A**, Figure 1). The latter one was first isolated from *Bacillus pumilus*, and shows *in vitro* cytotoxicity against human malignant A375-S2 and cervical cancer HeLa cells [4]. The paraphaeosphaerins (**B**) from cultures of *Paraphaeosphaeria quadriseptana* [5] are biogenetically related to the cytotoxic plant metabolites monocillin I and radicicol. NM-3 (**C**) is a synthetic analogue of cytogenin (**E**), and potentiates antineoplastic effects of other chemotherapeutic agents and inhibits angiogenesis [6]. This compound is in phase I clinical trials. The isocoumarin 185322 (**D**), an analogue of NM-3, is an inhibitor of microtubule assembly, and induces mitotic arrest and apoptosis of multiple myeloma cells [7]. Ochratoxin A (**F**), a mycotoxin from *Aspergillus ochraceus* shows nephrotoxic, hepatotoxic, carcinogenic and teratogenic properties in animals [8]; recently the European Pharmacopoeia introduced a limit test for ochratoxin A in plant material (Method 2.8.22). Scoparine A (**G**) was isolated from the North African medical plant *Pituranthos scoparius* [9].

The biological activities of the abovementioned and other isocoumarins and dihydroisocoumarins [10, 11] make this class of compounds interesting leads for development of new anticancer drugs.

In a previous work [12] we prepared a number of isocoumarins and dihydroisocoumarins bearing alkyl and hydroxyalkyl residues at C-3 using a one-pot procedure originally described by Lia and Cheng [13]. Since these products showed only moderate cytotoxic activities, we assumed that additional functional groups at the bicyclic core should be of great importance. An isocoumarin with an isopentyl residue at C-3 was the most cytotoxic one in the previous investigations. This prompted us to investigate the influence of additional lipophilic substituents like halogens (cf. **F**) and methyl (cf. **D**) located at various positions of the ring system on the cytotoxicity of 3-alkyl(dihydro)isocoumarins.

## Results and Discussion

### Chemistry

The isocoumarins **2a–2d** were prepared in poor to good yields in one pot reactions starting from commercially available 2-iodobenzoic acid derivatives **1a–d** and hept-1-yne involving Sonogashira cross coupling reactions in the presence of zinc chloride [13]. The isocoumarins **2a**, **2b** [14] and **2d** were further hydrogenated under palladium catalysis to give the corresponding dihydroisocoumarins **3a**, **3b** and **3d** (Scheme 1). Hydrogenation of **2c** did not appear promising due to the risk of reductive dechlorination.

The 4-chloroisocoumarin **5** was prepared in a different manner. Methyl 2-iodobenzoate (**1e**) was reacted in a Sonogashira reaction with hept-1-yne (in the absence of zinc chloride) [15, 16] to give the alkyne **4**. Treating **4** with 2 equivalents of CuCl_2_ and a catalytic amount of *N*,*N*-dicyclohexylammonium chloride [17] resulted in cyclization to the chlorinated isocoumarin **5** (Scheme 2).

### Biology

The resulting isocoumarins and dihydroisocoumarins were tested for their cytotoxic activity in a MMT assay using the HL 60 leukemia cell line [18, 19]. The results are shown in Table 1. Since we had found some correlation of the cytotoxic activities with lipophilicity in previous investigations on isocoumarins [12], we also calculated the log P values of the new compounds.

## Discussion

Among the five isocoumarins and three dihydroisocoumarins described here, four compounds, the fluorinated isocoumarin **2b** and the dihydroisocoumarins **3a**, **3b** and **3d**, showed measurable, albeit very poor cytotoxic activity. No clear tendency for higher acitivity of one of the two structural subtypes (**2** versus **3**) can be deduced from these results. Since the log P values of all tested compounds are in the same range, influences of lipophilicity on cytotoxic activity cannot be deduced as well.

In conclusion, isocoumarins and dihydroisocoumarins containing exclusively lipophilic residues at both rings show significantly decreased cytotoxic activities compared to established analogues (AI-77-B (**A**): 0.2 to 0.4 μM against A375-S2 and HeLa cells [4], ochratoxin A (**F**) and stereoisomers thereof: 0.3 to 5 μM against Hep G2 cells [20]) containing additional polar groups (especially hydroxyl groups) at the benzenoid ring.

For obtaining isocoumarins with significant cytotoxicity the presence of polar groups at the ring system seems to be indispensible.

## Experimental

Elemental analysis: Heraeus CHN Rapid; IR spectra: Perkin-Elmer FT-IR Paragon 1000; MS (EI, 70 eV): Hewlett Packard MS-Engine; HRMS: Jeol Mstation 700; NMR: Jeol GSX 400 (^1^H: 400 MHz, ^13^C: 100 MHz); flash column chromatography (FCC): Kieselgel 60 (230–400 mesh, E. Merck, Darmstadt).

### General procedure I: Synthesis of isocoumarins 2a–d

Pd(PPh_3_)_2_Cl_2_ (0.140 g, 0.20 mmol) and ZnCl_2_ (0.496 g, 4.0 mmol) were added under N_2_ atmosphere to a solution of aryl iodide (2.0 mmol), hept-1-yne (0.577 g, 6.0 mmol), and triethylamine (10 mmol) in DMF (2 mL). The suspension was heated at 100 °C for 24 h. The components of the suspension were separated by flash column chromatography (*n*-hexane/ethyl acetate).

### General procedure II: Preparation of dihydroisocoumarins 3a,b,d

Pd on charcoal (10%) was added to a solution of the isocoumarin in methanol (20 mL). The suspension was stirred for 12 h under H_2_ atmosphere at ambient pressure, then the catalyst was filtered off, and the residue was washed with methanol. The combined organic layers were evaporated, and the residue was purified by FCC (*n*-hexane/ethyl acetate).

#### 3-Pentylisochromen-1-one (**2a**)

Prepared following General procedure I from 0.496 g (2.0 mmol) 2-iodobenzoic acid to give 310 mg (71 %) as a pale yellow oil. MS (*m/z*, %): 216 ([M]^+·^, 34), 160 (36), 118 (100). IR (KBr), ν (cm^−1^): 2933, 2857, 1716, 1652, 1459, 1176, 769, 705, 592. ^1^H NMR (400 MHz, CDCl_3_): δ (ppm) 0.90 (t, *J* = 7.5 Hz, 3 H, H-5′), 1.35 (m, 4 H, H-3′, H-4′), 1.70 (m, 2 H, H-2′), 2.51 (t, *J* = 7.5 Hz, 2 H, H-1′), 6.26 (s, 1 H, H-4), 7.34 (d, *J* = 8.1 Hz, 1 H, H-5), 7.43 (dd, *J_1_* *= J_2_* = 8.1 Hz, 1 H, H-7), 7.66 (dd, *J_1_* *= J_2_* = 8.1 Hz, 1 H, H-6), 8.24 (d, *J* = 8.1 Hz, 1 H, H-8). ^13^C NMR (100 MHz, CDCl_3_): δ (ppm) 13.92 (C-5′), 22.34 (C-4′), 26.55 (C-2′), 31.13 (C-3′), 33.45 (C-1′), 102.83 (C-4), 120.06 (C-8a), 127.48 (C-7), 129.45 (C-5), 129.46 (C-8), 134.67 (C-6), 137.60 (C-4a), 158.28 (C-3), 163.12 (C-1). HRMS: Calcd. for C_14_H_16_O_2_: 216.1150. Found: 216.1100.

#### 8-Fluoro-3-pentyl-1*H*-isochromen-1-one (**2b**) [14]

Prepared following General procedure I from 0.532 g (2.0 mmol) 6-fluoro-2-iodobenzoic acid to give 39 mg (9 %) as a brown oil. MS (*m/z*, %): 234 ([M]^+·^, 19), 178 (24), 136 (100), 107 (30). IR (KBr), ν (cm^−1^): 2956, 2929, 2859, 1731, 1654, 1600, 1562, 1469, 1419, 1378, 1321, 1272, 1240, 1201, 1149, 1087, 1054, 1031, 971, 923, 894, 831, 773, 719, 678, 651, 570, 557. ^1^H NMR (400 MHz, CDCl_3_) δ (ppm): 0.91 (t, *J* = 7.7 Hz, 3 H, H-5′), 1.35 (m, 4 H, H-3′, H-4′), 1.70 (m, 2 H, H-2′), 2.50 (t, *J* = 7.7 Hz, 2 H, H-1′), 6.23 (d, *J* = 2.3 Hz, 1 H, H-4), 7.12 (m, 2 H, H-5, H-7), 7.61 (m, 1 H, H-6). HRMS: Calcd. for C_14_H_15_O_2_F: 234.1056. Found: 234.10594. Anal. Calcd. for C_14_H_15_O_2_F: C, 71.78; H, 6.45. Found: C, 72.27; H, 7.66.

#### 6-Chloro-3-pentyl-1*H*-isochromen-1-one (**2c**)

Prepared following General procedure I from 0.565 g (2.0 mmol) 4-chloro-2-iodobenzoic acid to give 75 mg (15 %) as a brown oil. MS (*m/z*, %): 252 ([M]^+·^, 13), 250 ([M]^+·^, 35), 194 (45), 152 (100), 123 (20). IR (KBr), ν (cm^−1^): 2956, 2929, 2859, 1774, 1745, 1660, 1614, 1567, 1475, 1376, 1315, 1253, 1157, 1105, 1012, 831, 808, 777, 728, 688, 669. ^1^H NMR (400 MHz, CDCl_3_): δ (ppm) 0.91 (t, *J* = 7.6 Hz, 3 H, H-5′), 1.35 (m, 4 H, H-3′, H-4′), 1.70 (m, 2 H, H-2′), 2.52 (t, *J* = 7.6 Hz, 2 H, H-1′), 6.18 (s, 1 H, H-4), 7.34 (d, *J* = 2.0 Hz, 1 H, H-5), 7.39 (dd, *J_1_* = 8.5 Hz, *J_2_* = 2.0 Hz, 1 H, H-7), 8.17 (d, *J* = 8.5 Hz, 1 H, H-8). ^13^C NMR (100 MHz, CDCl_3_): δ (ppm) 13.94 (C-5′), 22.35 (C-4′), 26.50 (C-2′), 31.14 (C-3′), 33.55 (C-1′), 102.02 (C-4), 118.41 (C-8a), 124.56 (C-5), 128.02 (C-7), 131.20 (C-8), 139.00 (C-4a), 141.34 (C-6), 159.86 (C-3), 162.30 (C-1). HRMS: Calcd. for C_14_H_15_O_2_Cl: 250.0761. Found: 250.0834. Anal. Calcd. for C_14_H_15_O_2_Cl: C, 67.07; H, 6.03. Found: C: 67.93; H: 6.90.

#### 5-Methyl-3-pentyl-1H-isochromen-1-one (**2d**)

Prepared following General procedure I from 0.524 g (2.0 mmol) 2-iodo-3-methylbenzoic acid to give 80 mg (18 %) as a yellow oil. MS (*m/z*, %): 230 ([M]^+·^, 48), 174 (37), 132 (100), 103 (13). IR (KBr), ν (cm^−1^): 2927, 2857, 1724, 1652, 1465, 1380, 1270, 1174, 1058, 1022, 759, 705, 584. ^1^H NMR (400 MHz, CDCl_3_): δ (ppm) 0.81 (t, *J* = 7.6 Hz, 3 H, H-5′), 1.29 (m, 4 H, H-3′, H-4′), 1.65 (m, 2 H, H-2′), 2.39 (s, 3 H, Ar-CH_3_), 2.47 (t, *J* = 7.6 Hz, 3 H, H-1′), 6.29 (s, 1 H, H-4), 7.26 (dd, *J_1_* *= J_2_* *=* 7.8 Hz, 1 H, H-7), 7.44 (d, *J* = 7.8 Hz, 1 H, H-6), 8.05 (d, *J* = 7.8 Hz, 1 H, H-8). ^13^C NMR (100 MHz, CDCl_3_): δ (ppm) 13.96 (C-5′), 18.72 (Ar-CH_3_), 22.45 (C-4′), 26.78 (C-2′), 31.21 (C-3′), 33.86 (C-1′), 99.66 (C-4), 120.12 (C-4a), 127.00 (C-7), 127.36 (C-8), 132.57 (C-8a), 135.90 (C-6), 136.32 (C-5), 157.89 (C-3), 163.52 (C-1). HRMS: Calcd. for C_15_H_18_O_2_: 230.1307. Found: 230.1317. Anal. Calcd. for C_15_H_18_O_2_: C, 77.38; H, 9.74. Found: C: 77.17; H, 8.03.

#### (±)-3-Pentylisochroman-1-one (*rac*-(3*R*)-3-pentyl-3,4-dihydro-1*H*-isochromen-1-one, **3a**)

Following General procedure II from 200 mg (0.925 mmol) 3-pentylisochromen-1-one (**2a**) using 20 mg Pd on charcoal (10 %) to give 160 mg (80 %) as a colourless oil. MS (*m/z*, %): 218 ([M]^+·^, 12), 147 (72), 118 (100), 105 (28). IR (KBr), ν (cm^−1^): 2943, 2855, 1705, 1650, 1455, 1170, 765, 701, 593. ^1^H NMR (400 MHz, CDCl_3_): δ (ppm) 0.91 (t, *J* = 7.2 Hz, 3 H, H-5′), 1.34 (m, 4 H, H-3′, H-4′), 1.47 (m, 1 H, H-2′), 1.58 (m, 1 H, H-2′), 1.72 (m, 1 H, H-1′), 1.88 (m, 1 H, H-1′), 2.94 (m, 2 H, H-4), 4.52 (m, 1 H, H-3), 7.24 (dd, *J_1_* *= J_2_* = 7.6 Hz, 1 H, H-5), 7.38 (dd, *J_1_* *= J_2_* = 7.6 Hz, 1 H, H-6), 7.52 (ddd, *J_1_* = *J_2_* = 7.6 Hz, *J_3_=* 1.4 Hz, 1 H, H-7), 8.09 (d, *J* = 7.6 Hz, 1 H, H-8). ^13^C NMR (100 MHz, CDCl_3_): δ (ppm) 14.01 (C-5′), 22.53 (C-4′), 24.60 (C-2′), 31.57 (C-3′), 33.21 (C-4), 34.94 (C-1′), 77.39 (C-3), 125.23 (C-4a), 127.37 (C-5), 127.58 (C-6), 130.23 (C-8), 133.64 (C-7) 139.25 (C-8a), 165.74 (C-1). HRMS: Calcd. for C_14_H_18_O_2_: 218.1307. Found: 218.1286. Anal. Calcd. for C_14_H_18_O_2_: C, 77.38; H, 9.74. Found: C, 77.17; H, 8.03.

#### (±)-8-Fluoro-3-pentylisochroman-1-one (*rac*-(3*R*)-8-fluoro-3-pentyl-3,4-dihydro-1*H*-isochromen-1-one, **3b**)

Following General procedure II from 28 mg (0.12 mmol) 8-fluoro-3-pentylisochromen-1-one (**2b**) using 5 mg Pd on charcoal (10 %) to give 25 mg (89 %) as a pale yellow oil. IR (KBr), ν (cm^−1^): 3456, 2955, 2928, 2858, 1731, 1660, 1615, 1582, 1470, 1378, 1358, 1253, 1099, 1057, 1031, 803, 780, 727, 690. ^1^H NMR (400 MHz, CDCl_3_): δ (ppm) 0.91 (t, *J* = 7.1 Hz, 3 H, H-5′), 1.33 (m, 4 H, H-3′, H-4′), 1.45 (m, 1 H, H-2′), 1.57 (m, 1 H, H-2′), 1.71 (m, 1 H, H-1′), 1.87 (m, 1 H, H-1′), 2.94 (m, 2 H, H-4), 4.48 (m, 1 H, H-3), 7.09 (m, 2 H, H-5 and H-7), 7.50 (m, 1 H, H-6). ^13^C-NMR (125 MHz, CDCl_3_) δ (ppm): 14.04 (C-5′), 22.53 (C-4′), 24.55 (C-2′), 31.58 (C-4), 33.65 (d, *J* = 2.4 Hz, C-4), 34.73 (C-1′), 78.53 (C-3), 113.87 (d, *J* = 6.9 Hz, C-8a), 116.01 (d, *J* = 21.6 Hz, C-7), 123.07 (d, *J* = 3.8 Hz, C-5), 134.95 (d, *J* = 10.0 Hz, C-6), 141.90 (C-4a), 161.43 (C-1), 162.99 (d, *J* = 264.8 Hz, C-8). HRMS: Calcd. for C_15_H_20_O_2_: 236.1213. Found: 236.1195.

#### (±)-5-Methyl-3-pentyl-isochroman-1-one (*rac*-(3*R*)-5-methyl-3-pentyl-3,4-dihydro-1*H*-isochromen-1-one, **3d**)

Following General procedure II from 40 mg (0.17 mmol) 5-methyl-3-pentylisochromen-1-one (**2d**) using 4 mg Pd on charcoal (10 %) to give 35 mg (89 %) as a colourless oil. MS (*m/z*, %): 232 ([M]^+·^, 21), 161 (63), 133 (100), 105 (29). IR (KBr), ν (cm^−1^): 2956, 2929, 2859, 1774, 1745, 1660, 1614, 1567, 1475, 1376, 1315, 1253, 1157, 1105, 1012, 831, 808, 777, 728, 688, 669. ^1^H NMR (400 MHz, CDCl_3_): δ (ppm) 0.91 (t, *J* = 7.0 Hz, 3 H, H-5′), 1.34 (m, 4 H, H-3′, H-4′), 1.49 (m, 1 H, H-2′), 1.59 (m, 1 H, H-2′), 1.74 (m, 1 H, H-1′), 1.89 (m, 1 H, H-1′), 2.32 (s, 3 H, Ar-CH_3_), 2.77 (dd, *J_1_* = 3.3 Hz, *J_2_* = 16.6 Hz, 1 H, H-4), 2.92 (dd, *J_1_* = 3.3 Hz, *J_2_* = 16.6 Hz, 1 H, H-4), 4.48 (m, 1 H, H-3), 7.27 (dd, *J_1_* = *J_2_* = 7.7 Hz, 1 H, H-7), 7.39 (d, *J* = 7.7 Hz, 1 H, H-6), 7.96 (d, *J* = 7.7 Hz, 1 H, H-8). ^13^C-NMR (125 MHz, CDCl_3_) δ (ppm): 13.98 (C-5′), 18.85 (Ar-CH_3_), 22.49 (C-4′), 24.62 (C-2′), 30.23 (C-4), 31.57 (C-3′), 35.08 (C-1′), 77.25 (C-3), 125.20 (C-4a), 126.93 (C-7), 128.07 (C-8), 134.94 (C-6), 135.00 (C-8a), 137.78 (C-5), 166.08 (C-1). HRMS: Calcd. for C_15_H_20_O_2_: 232.14633. Found: 232.14310.

#### Methyl 2-(hept-1-yn-1-yl)benzoate (**4**)

2.62 g (10.0 mmol) methyl 2-iodobenzoate, 0.96 g (10.0 mmol) hept-1-yne, 200 mg (2.4 mmol) CuI and 400 mg (0.6 mmol) PdCl_2_(PPh_3_)_2_ were dissolved in 20 mL *N*-ethyl-*N*,*N*-dimethylamine and the solution was stirred for 12 h at room temperature. The solvent was evaporated and the residue was quenched with 20 mL 5% aqueous Na_2_SO_3_-solution. The mixture was extracted with diethyl ether (3 x 25 mL) and the combined organic layers were dried over Na_2_SO_4_. The solvent was evaporated and the residue was purified by FCC (isohexane/ethyl acetate 5:1) to give 740 mg (32 %) of **4** as a pale brown oil. MS (*m/z*, %): 230 ([M]^+·^, 22), 215 (36), 174 (100), 159 (72), 133 (96), 115 (42). IR (KBr), ν (cm^−1^): 3066, 2954, 2931, 2859, 2235, 1733, 1596, 1565, 1484, 1446, 1432, 1294, 1274, 1249, 1130, 1083, 962, 881, 757, 701, 538. ^1^H-NMR (400 MHz, CDCl_3_) δ (ppm): 0.90 (t, *J* = 7.2 Hz, 3 H, H-7′), 1.35 (m, 2 H, H-5′), 1.43 (m, 2 H, H-6′), 1.62 (m, 2 H, H-4′), 2.44 (t, *J* = 7.2 Hz, 2 H, H-3′), 3.88 (s, 3 H, OCH_3_), 7.27 (ddd, *J_1_= J_2_* = 7.8 Hz, *J_3_* = 1.2 Hz, 1 H, H-4), 7.38 (ddd, *J_1_= J_2_* = 7.8 Hz, *J_3_* = 1.2 Hz, 1 H, H-5), 7.48 (dd, *J_1_* = 7.8 Hz, *J_2_* = 1.2 Hz, 1 H, H-3), 7.85 (dd, *J_1_* = 7.8 Hz, *J_2_* = 1.2 Hz, 1 H, H-6). ^13^C NMR (100 MHz, CDCl_3_): δ (ppm) 13.91 (C-7′), 19.66 (C-6′), 22.16 (C-4′), 28.31 (C-5′), 31.03 (C-3′) 51.93 (OCH_3_), 79.10 (C-1′), 95.95 (C-2′), 124.50 (C-2), 127.00 (C-4), 130.03 (C-5), 131.36 (C-3), 131.38 (C-1), 134.10 (C-6), 166.91 (C=O). Anal. Calcd. for C_15_H_18_O_2_: C, 78.26; H, 7.83. Found: C, 78.69; H, 7.76.

#### 4-Chloro-3-pentyl-1*H*-isochromen-1-one (**5**)

0.690 g (3.20 mmol) methyl 2-(hept-1-ynyl)-benzoate (**4**), 0.081 g (6.2 mmol) CuCl_2_, 0.065 g (0.30 mmol) *N*,*N*-dicyclohexylammonium chloride and 30 mL 1,2-dichloroethane were dissolved under N_2_ atmosphere and heated at 80 °C with stirring. After 12 h the solvent was evaporated and the residue was purified by FCC (isohexane/ethyl acetate 10:1) to give 580 mg (73 %) of **5** as a pale brown oil. MS (*m/z*, %): 252 ([M]^+·^, 31), 250 ([M]^+·^, 100), 215 (18), 194 (69), 165 (26), 159 (51), 152 (43). IR (KBr), ν (cm^−1^): 3072, 3035, 2956, 2931, 2859, 1745, 1631, 1602, 1567, 1479, 1465, 1319, 1288, 1052, 1031, 975, 964, 925. ^1^H NMR (400 MHz, CDCl_3_): δ (ppm) 0.90 (t, *J* = 7.7 Hz, 3 H, H-5′), 1.37 (m, 4 H, H-3′, H-4′), 1.73 (m, 2 H, H-2′), 2.75 (t, *J* = 7.7 Hz, 2 H, H-1′), 7.53 (ddd, *J_1_= J_2_* = 7.8 Hz, *J_3_* = 1.2 Hz, 1 H, H-7), 7.79 (m, 2 H, H-5, H-6), 8.26 (d, *J* = 7.8 Hz, 1 H, H-8). ^13^C NMR (100 MHz, CDCl_3_): δ (ppm) 13.89 (C-5′), 22.30 (C-4′), 26.41 (C-2′), 31.17 (C-1′, 3′), 110.89 (C-4), 120.13 (C-8a), 123.14 (C-5), 128.35 (C-7), 129.73 (C-8), 135.17 (C-6), 135.54 (C-4a), 154.66 (C-3), 161.31 (C-1). Anal. Calcd. for C_14_H_15_O_2_Cl: C, 67.07; H, 6.03. Found: C, 67.24; H, 6.45.

### MTT assay

A solution of the substance in dimethyl sulfoxide (1 μl, concentrations in the range from 10^−9^ to 10^−4^ mol/l) was incubated with 99 μl of a suspension of HL 60 cells (9 × 10^5^ cells/ml) in RPMI 1640 medium (PAA Laboratories) with 10% FKS in 96 well plates for 24 h. Then, 10 μl of a solution of MTT (3-(4,5-dimethylthiazol-2-yl)-2,5-diphenyltetrazolium bromide) in PBS (5 mg/ml) were added and the plate was incubated for another 2 h. The cells were quenched with 190 μl dimethyl sulfoxide and after a few min, the plates were evaluated on a Dynatech MRX at a wavelength of 570 nm; the reference wavelength was 630 nm [18]. The experiments were performed in triplicate.

## Figures and Tables

**Sch. 1.**
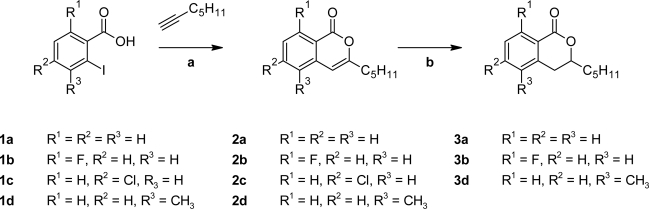
a: Triethylamine, ZnCl_2_, Pd(PPh_3_)_2_Cl_2_, DMF, 100 °C, 24 h ; b: H_2_, Pd/C, methanol, 25 °C, 12 h.

**Sch. 2.**
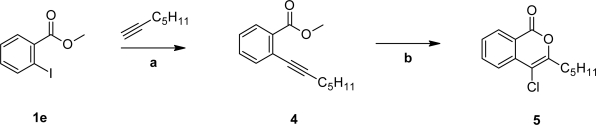
a: Pd(PPh_3_)_2_Cl_2_, CuI, *N*-ethyl-*N*,*N*-dimethylamine, DMF, 25 °C, 12 h; b: CuCl_2_, *N*,*N*-dicyclohexylammonium chloride, 1,2-dichloroethane, 80 °C, 12 h.

**Tab. 1.** Cytotoxicity of the products against HL 60 cell line and calculated partition coefficients log P (calculated with Chem Draw Ultra 10, Cambridge Soft).

**Compound**	**IC_50_ [μM]**	**log P**
**2a**	≥100	3.32
**2b**	58	3.48
**2c**	≥100	3.88
**2d**	≥100	3.81
**3a**	49	3.89
**3b**	63	3.48
**3d**	103	4.38
**4**	>1000	4.28
**5**	≥100	4.74
**cisplatin**	5	
